# Round-robin differential-phase-shift quantum key distribution with a passive decoy state method

**DOI:** 10.1038/srep42261

**Published:** 2017-02-13

**Authors:** Li Liu, Fen-Zhuo Guo, Su-Juan Qin, Qiao-Yan Wen

**Affiliations:** 1 State Key Laboratory of Networking and Switching Technology, Beijing University of Posts and Telecommunications, Beijing, 100876, China; 2 School of Science, Beijing University of Posts and Telecommunications, Beijing, 100876, China

## Abstract

Recently, a new type of protocol named Round-robin differential-phase-shift quantum key distribution (RRDPS QKD) was proposed, where the security can be guaranteed without monitoring conventional signal disturbances. The active decoy state method can be used in this protocol to overcome the imperfections of the source. But, it may lead to side channel attacks and break the security of QKD systems. In this paper, we apply the passive decoy state method to the RRDPS QKD protocol. Not only can the more environment disturbance be tolerated, but in addition it can overcome side channel attacks on the sources. Importantly, we derive a new key generation rate formula for our RRDPS protocol using passive decoy states and enhance the key generation rate. We also compare the performance of our RRDPS QKD to that using the active decoy state method and the original RRDPS QKD without any decoy states. From numerical simulations, the performance improvement of the RRDPS QKD by our new method can be seen.

Quantum key distribution (QKD) enables two distant parties (Alice and Bob) to share a key, which is secret from any eavesdropper (Eve)^1^. It has been proved to be unconditional secure theoretically^2^. QKD has been widely studied in both theoretical and experimental research^3^,^4^ since its initial proposal. Moreover, QKD has entered the commercial market^5^ and small QKD networks have been realized^6^.

Since the rise of the BB84 protocol^1^, many QKD protocols have been proposed^7^,^8^,^9^,^10^,^11^. The security proofs of QKD focus on how much the information is leaked to Eve. The information leakage generally can be estimated through monitoring some statistics by Alice and Bob^2^,^12^,^13^,^14^,^15^,^16^. The conventional QKD protocols inherently rely on the original version of Heisenberg’s uncertainty principle, which dictates that the more information Eve has obtained, the more disturbance she should have caused on the signal. Recently, a new type of protocol, called round-robin differential-phase-shift (RRDPS) QKD protocol^17^, was proposed and surprisingly, the information leakage of this protocol is estimated without any monitoring, but depends only on the state prepared by Alice. The RRDPS QKD protocol has higher stability and lower loss, it can also tolerate more noisy channels^18^,^19^. Since the RRDPS QKD was proposed, it has been studied both theoretically^18^,^19^,^20^ and experimentally^20^,^21^,^22^,^23^.

Unfortunately, due to the imperfections of devices, there is still a big gap between the theory and practice of QKD. The decoy state method has been used in the general BB84 protocol^24^,^25^,^26^,^27^,^28^,^29^ to defeat the photon-number-splitting (PNS) attack^29^,^30^ and guarantee the security against imperfect sources, such as weak coherent pulses (WCPS)^27^. Recently, a tight bound on the key rate of RRDPS QKD was given in ref. 18, in which it was also proposed that the infinite decoy state method for RRDPS QKD would improve the key rate. Ying-Ying Zhang *et al*.^31^ extended it to the practical case with a finite number of decoy states and got the performance close to the infinite decoy state method. These approaches are all related to the active decoy state selection, which is based on the assumption that Eve can not distinguish decoy and signal states. However, this assumption may not stand in real active decoy state experiments, for which it may open up to side channel attacks and even break the security of the system when one actively modulates the intensities of pulses^32^,^33^. The passive decoy state method^34^,^35^,^36^,^37^ can reduce the side channel information in the decoy state preparation procedure. Different from the active decoy state method, the passive one only uses one intensity signal, and Alice post-selects the signal state and the decoy state according to the response of Alice’s own detector. The method in ref. 36 extended passive decoy state to practical unstable light sources including phase-randomized WCPs, which inspired its application to practical QKD.

In this paper, we apply the passive decoy state method to the RRDPS QKD protocol. Alice uses weak coherent sources with random phases to passively generate signal states or decoy states. Not only can the more environment disturbance be tolerated, but also one can avoid the side channel attacks on sources, which may be generated by active modulation of source intensities. Most of all, we apply a strategy that gives the accurate probability of having 0, 1, 2 photons and omits the other multiphoton occurrences. Our method is accordant with practical systems. we also show the performance comparison between our method, the active decoy state method and the original RRDPS protocol in our paper. A performance improvement of our RRDPS QKD using passive decoy state method can be seen in numerical simulations. It shows that under the same key generation rate, our protocol will have longer transmission distance.

## Results

### RRDPS QKD with passive decoy state strategy

**Figure 1:**
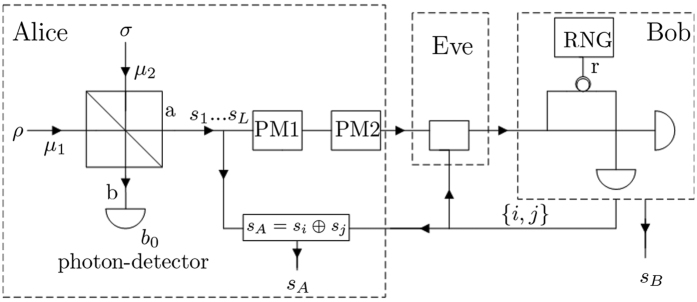
Basic setup of a passive decoy state RRDPS QKD scheme: interference of two Fock diagonal states, *ρ* and *σ*, at a beam splitter (BS) of transmittance *t*; *a* and *b* represent the two output modes. Signals flow through lines. PM 1 adds a random phase on each pulse train, and PM 2 encodes random phases 0 or *π* on each pulse. Eve tries to guess Alice’s bit *s*_*A*_ = *s*_*i*_ ⊕ *s*_*j*_ in the figure, where indices {*i, j*} are announced by Bob. According to the random number generator RNG, Bob conducts measurement to guess *s*_*A*_.

In this section, we apply the passive decoy state method to the RRDPS QKD protocol^17^, as shown in Fig. 1.

The protocol proceeds as follows:

1. Alice uses two weak coherent pulses with random phases to passively generate signal or decoy states. In this way she can prepare a series of pulse trains with each contains *L* pulses, and each train encodes a random *L*-bit sequence *s* = (*s*_1_*s*_2_...*s*_*L*_) on a weak signal. Then she applies phase modulation {0, *π*} to each optical mode according to *s* and obtains the state 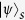 as in Eq. (1),
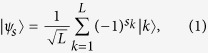
where the photon is in the *k*-th pulse for state 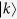, *s*_*k*_ is the encoded bit sequence. She sends 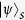 to Bob.
2. Bob splits the received signal with a 50/50 beam splitter to obtain two *L*-pulse trains, uses RNG to generate a random number *r* ∈ {−*L* + 1, …, −2, −1, 1, 2, …, *L* − 1}, and shifts one of the *L*-pulse trains forward (*r* > 0) or backward (*r* < 0) by |*r*| pulses.
3. Bob measures the interference between two *L*-pulse trains. If he obtains a detection on position *i* in the unshifted pulse train, corresponding to position *j* in the shifted pulse train, and 0 ≤ *j* = *i* + *r* ≤ *L* − 1, Bob records a raw key bit according to the relative phase *s*_*B*_ = *s*_*i*_ ⊕ *s*_*j*_. Otherwise, Bob regards the transmission as a failure.
4. Bob announces {*i, j*} so that Alice can obtain the sifted key, *s*_*A*_ = *s*_*i*_ ⊕ *s*_*j*_.

Alice generates phase-randomized pulses using two weak coherent sources with intensities *μ*_1_ and *μ*_2_ per pulse, respectively. It passively generates signal and decoy states, which is a joint-distribution state according to the result of detector *b*_0_. *ρ* and *σ* denote the coherent states of two phase-randomized WCP sources states, respectively,


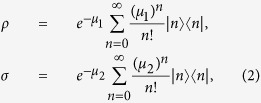


with *μ*_1_ and *μ*_2_ denoting the mean photon number of the two signals. The joint probability of having *n* photons in output mode a and *m* photons in output mode b can be written as ref. 38


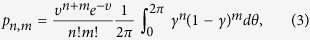


where the parameters *υ, γ* and *θ* are given by


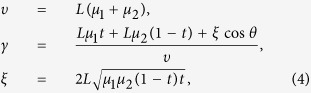


and *L* denotes the number of pules, *t* denotes the transmittance of a beam splitter. This result differs from the one expected from the interference of two pure coherent states with fixed phase relation, 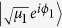 and 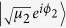, at a BS of transmittance *t*. In this last case, *p*_*n,m*_ is just the product of two Poissonian distributions.

When Alice ignores the outcome of the measurement in mode b, the probability of having *n* photons in mode a can be written as


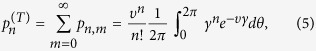


which is proven to be a non-Poissonian probability distribution^38^ and 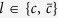.

For Alice’s detector *b*_0_, the joint probability of having *n* photons in mode a and no click in the threshold detector *b*_0_ has now the form


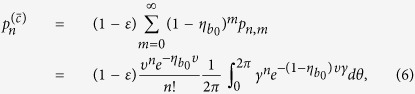


where the parameter *ε* denotes dark count and 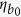 denotes the single photon detection efficiency of the detector. 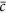 indicates the detector *b*_0_ has no click. Then, the probability of having *n* photons in mode a and producing a click in Alice’s threshold detector *b*_0_ is


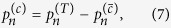


where *c* indicates the detector *b*_0_ has a click.

### Estimation of the key generation rate

We modify the Gottesman-Lo-Lutkenhaus-Preskill (GLLP) formula^39^ according to the RRDPS QKD security analysis^18^. From the GLLP formula, we have


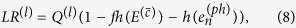


where *R*^(*l*)^, 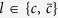 indicates the key generation rate of RRDPS QKD with passive decoy state between Alice and Bob. 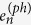 denotes the phase error rate of *n*-photon pulses. *f* is the efficiency of the error correction protocol, 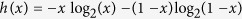 is the binary Shannon entropy function. *Q*^(*l*)^ and *E*^(*l*)^ indicate the total gain and the quantum bit error rate (QBER) corresponding to setting *l*, respectively. Thus, combine with 
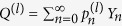
, we get the new key generation rate formula


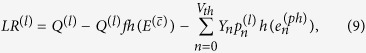


where we denote the output that cause no click of Alice’s detector *b*_0_ as signal states. The ones that cause a click of Alice’s detector *b*_0_ are decoy states. As for RRDPS protocol, the phase error rate depends on the preparation of quantum states rather than the transmission process. When the number of photons in a train is no more than an integer 
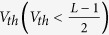
, the phase error rate 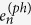 can be bounded by *V*_*th*_/*L* − 1^13^. So we can get *R*, the final key generation rate per pulse of RRDPS QKD with passive decoy state between Alice and Bob, it’s the main parameter to evaluate the performance of protocol,


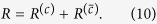


Next, we give how to obtain the parameters *Q*^(*l*)^ and *E*^(*l*)^ corresponding to setting *l*. The gain *Q*^(*l*)^ corresponding to setting *l* is the probability that Bob obtains a click in his measurement apparatus when Alice sends him a state prepared with setting *l*. It can be written as


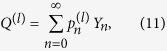


where *Y*_*n*_ denotes the yield of an *n*-photon state. Similarly, the quantum bit error rate (QBER) associated to setting *l*, which we shall denote as *E*^(*l*)^, is given by


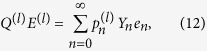


with *e*_*n*_ representing the error rate of an *n*-photon state.

The yields *Y*_*n*_ can be expressed as refs 34,35


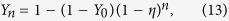


where *Y*_0_ is the background rate, *η* represents the overall transmittance of the system. This quantity can be written as


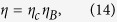


where *η*_*c*_ is the transmittance of the quantum channel, and *η*_*B*_ denotes the overall transmittance of Bob’s detection apparatus; that is, *η*_*B*_ includes the transmittance of any optical component within Bob’s measurement device and the detector efficiency. The parameter *η*_*c*_ can be related with a transmission distance *D* measured in km for the given QKD scheme as


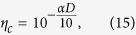


where *α* represents the loss coefficient of the channel measured in dB/km.

The *n*-photon error rate *e*_*n*_ is given by refs 25,26


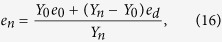


where *e*_*d*_ is the probability that a signal hits the wrong detector on Bob’s side due to the misalignment in the quantum channel and in his detection setup. As usual, we also consider that the background is random (i.e. *e*_0_ = 1/2).

*Q*^(*c*)^ and 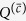 denote the overall gains in the case of Alice’s detector producing a click and no click, respectively. *Q*^(*T*)^ denotes the overall gain that Alice ignores the result of her measurement in mode *b*, i.e. the sum of the gains *Q*^(*c*)^ and 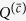. After substituting Eqs (5), (6) and (13, 14, 15) into the gain formulas Eq. (11) we obtain:


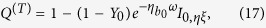



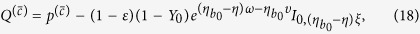



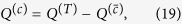


where *I*_*q,z*_ represents the modified Bessel function of the first kind, *ω* = *Lμ*_1_*t* + *Lμ*_2_(1 − *t*). From the Eqs (11), (12), (16) we can get:


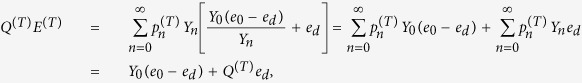


thus


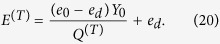


Then, in a similar way, we can get


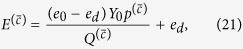



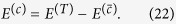


And from the Eq. (6) we have


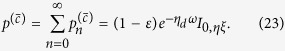


For practical implementations, large photon numbers are negligible comparing with those from small photon numbers. So we only consider the photon numbers for *n* = 0, 1, 2. The expressions of 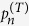 with *n* = 0, 1, 2 in Eq. (5) are ref. 37:


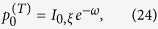



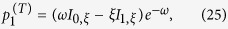



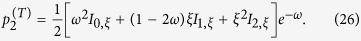


The probabilities 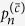 with *n* = 0, 1, 2 in Eq. (6) have the form ref. 37


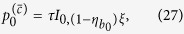



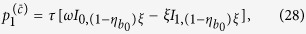



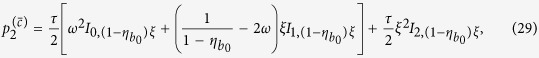


where 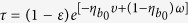.

### Numerical Simulation

**Figure 2:**
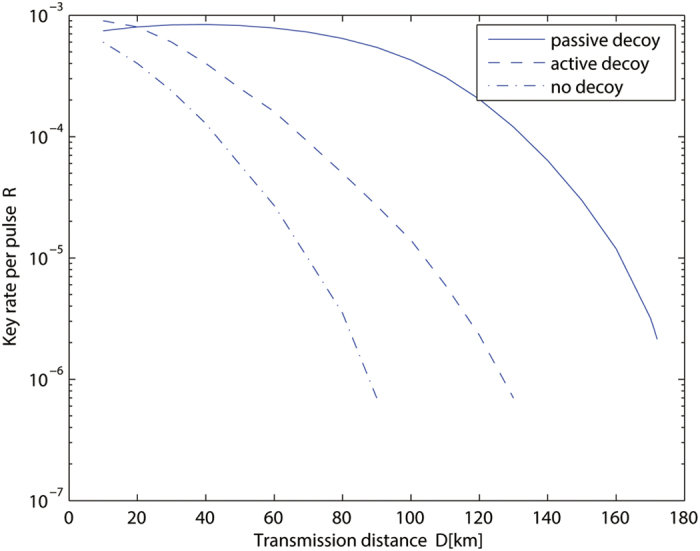
Key generation rate vs the transmission distance in RRDPS QKD with the passive decoy state method (solid line), the active decoy state method (dashed line; ref.^31^) and no decoy state (dot-dashed line; ref. 17).

According to the security analysis in above section, we can get the key generation rate Eq. (8) plotted in Fig. 2.

The parameters used in our method are the misalignment error rate *e*_*d*_ = 1.5%, the background rate *Y*_0_ = 3 × 10^−6^, *η*_*d*_ = 0.12, and *f* = 1, *t* = 1/2, which are the same as those in the original proposal for the active decoy state method in ref. 40. Then we can get


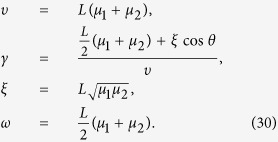


We show the relations between key generation rate and the transmission distance in RRDPS QKD protocol in Fig. 2. Given the certain transmission distance, we optimize the intensity of sources to maximize the key generation rate. According to the practical system, the key generation rate had better not lower than 10^7^. Thus we can obtain the maximal transmission distance as shown in 2.

From Fig. 2, we can see that the longer the transmission distance *D* is, the smaller the key generation rate *R* will be. We also show the performance comparison between our method, the active decoy state method^31^ and the original protocol RRDPS^17^. It can clearly be seen that the passive decoy state method can provide a performance improvement over the active one and the original one. That is, under the same key generation rate, our protocol will have longer transmission distance. Furthermore, it can defeat the photon-number-splitting (PNS) attack and guarantee the security against the imperfect sources compared to the original RRDPS QKD protocol^17^. It can also eliminate side channel attacks on sources, which may be caused by actively modulating decoy states^31^.

## Discussion

In summary, we apply the passive decoy state method in the RRDPS QKD which was proposed recently, and give a security analysis of this protocol. Using the passive decoy state method, the RRDPS QKD protocol provides a secure way to exchange private information without monitoring conventional disturbances and still maintains a high tolerance of noise. And it can also exclude the source side channel attacks, which the active source modulation method may bring. According to the RRDPS QKD security analysis, we modify the GLLP formula and derive a new key generation rate formula for our RRDPS protocol using passive decoy state method. Most importantly, we enhance the key generation rate. From the numerical simulations, we find that the RRDPS QKD with the passive decoy state method can have a performance improvement to the protocol with the active decoy state method and the original RRDPS protocol without decoy states.

The active decoy state method itself may introduce another loophole while closing the loophole of multiphoton pulses. As is well known, the active decoy state method is demonstrated based on the assumption that Eve can never distinguish the decoy state and the signal state. Unfortunately, this assumption is invalid in certain conditions, and Eve can beat the decoy state method due to the property of the intensity modulator. ref. 32 demonstrates that Eve can get full information about the key generated between the legitimate parties in QKD with active decoy state method. Compared with active selection, the passive decoy state method can reduce the side channel information in the decoy state preparation procedure. Thus, the passive signal and decoy state selection can avoid the side channel attacks on sources, which may be generated by active modulation of source intensities. Although the passive decoy state method can not remove all side channel attacks on sources, it can still avoid more attacks than the protocol with no decoy states and the active decoy states. Similar to the active decoy state method, the passive one can also defeat PNS attack. So we apply the passive decoy state method to the RRDPS QKD protocol, this strategy is very promising for applications of practical systems.

## Additional Information

**How to cite this article:** Liu, L. *et al*. Round-robin differential-phase-shift quantum key distribution with a passive decoy state method. *Sci. Rep.* **7**, 42261; doi: 10.1038/srep42261 (2017).

**Publisher's note:** Springer Nature remains neutral with regard to jurisdictional claims in published maps and institutional affiliations.
